# Holmes on Puerperal Fever

**Published:** 1855-03

**Authors:** 


### HOLMES ON PUERPERAL FEVER.

Dr. Oliver Wendell Holmes published, some years ago, a paper on the contagiousness of puerperal fever, the doctrines of which have been assailed in such a way as, in the judgment of the author, to call for the republication of his essay, a copy of which is before us.

The essay is prefaced by some remarks in reply to the criticism of Dr. Meigs, characterized by all the earnestness, pungency, and force for which Dr. Holmes is remarkable. We agree with him that the question is a grave and momentous one, not to be "smoothed over by nicely adjusted phrases of half-assent and half-censure divided between the parties," but to be answered clearly and emphatically. Dr. Holmes adds:—"If I have been hasty, presumptuous, ill-informed, illogical; if my array of facts means nothing; if there is no reason for any caution in the view of these facts; let me be told so, on such authority that I must believe it, and I will be silent henceforth, recognizing that my mind is in a state of disorganization. If the doctrine I have maintained is a mournful truth; if to disbelieve it, and to practise on this disbelief, and to teach others so to disbelieve and practise, is to carry desolation, and to charter others to carry it into confiding families, let it be proclaimed as plainly what is to be thought of the teachings of those who sneer at the alleged dangers, and scout the very idea of precaution. Let it be remembered that *persons* are nothing in this matter; better that twenty pamphleteers should be silenced, or as many professors unseated, than that one mother's life should be taken. There is no quarrel here between men, but there is deadly incompatibility and exterminating warfare between doctrines. *Coincidences*, meaning nothing, though a man have a monopoly of the disease for weeks or months; or *cause and effect*, the cause being in some way connected with the person; this is the question. If I am wrong, let me be put down by such a rebuke as no rash declaimer has received since there has been a public opinion in the medical profession of America; if I am right, let doctrines which lead to professional homicide be no longer taught from the chairs of those two great Institutions. Indifference will not do here; our Journalists and Committees have no right to take up their pages with minute anatomy and tediously detailed cases, while it is a question whether or not the 'black-death' of child-bed is to be scattered broadcast by the agency of the mother's friend and adviser. Let the men who mould opinions look to it; if there is any voluntary blindness, and interested oversight, any culpable negligence, even, in such a matter, and the facts shall reach the public ear; the pestilence-carrier of the lying-in chamber must look to God for pardon, for man will never forgive him."

This pamphlet is as powerful in argument as it is terse, clear, direct and vivid in its style. We earnestly recommend its perusal to every physician who proposes to engage in obstetrical practice, that he may see what can be urged of puerperal fever as a private pestilence.

